# Peach [*Prunus persica* (L.) Batsch] Cultivars Differ in Apparent Base Temperature and Growing Degree Hour Requirement for Floral Bud Break

**DOI:** 10.3389/fpls.2022.801606

**Published:** 2022-02-11

**Authors:** Douglas G. Bielenberg, Ksenija Gasic

**Affiliations:** ^1^Department of Biological Sciences, Clemson University, Clemson, SC, United States; ^2^Plant and Environmental Sciences, Clemson University, Clemson, SC, United States

**Keywords:** heat requirement, chilling requirement (CR), germplasm (genetic) resources, thermal time, bloom time

## Abstract

Bud break timing in peach [*Prunus persica* (L.) Batsch] is determined by the sequential fulfillment of a chilling requirement (CR) and a heat requirement (HR) for development. Genotypic variation in CR has been well characterized in peach. Adapting peaches to low chilling environment through reduced CR can make them susceptible to crop destroying spring frosts, if bloom occurs too early. Potential variation in HR between accessions has received less attention due to the methodological difficulty in assessing HR independently of CR. HR could vary in the magnitude of growing degree hours (GDHs) and/or the base temperature at which GDH accumulation begins. Characterizing HR traits in peach accessions could allow improved bloom time modeling and selection of phenotypes with improved spring frost avoidance through delayed bloom. We estimated GDH and apparent base temperature for floral bud break by observing time to floral bud break at several constant forcing temperatures. We evaluated 54 peach accessions (representing a range of CR) in which chilling had been saturated after >1,700 h at 3°C. Accessions differed widely in both the GDH requirement (2,015 to 11,191°C⋅h) and apparent base temperature (−1.85 to 8.69°C) for GDH accumulation. GDH and apparent base temperature were negatively correlated. A simulation exercise was performed to assess relative importance of varying base temperature vs. GDH for delaying bloom at different chilling accumulations at three locations in the southeastern United States using 30 years of historical weather data. The aim of this study was to determine whether there may be unrecognized diversity in peach germplasm for two HR traits (base temperature and thermal time) to enable breeding efforts to delay floral bud break and reduce the frost exposure risk of developing flowers and fruits. Our results suggest that selecting cultivars for increased GDH would be a safer, more reliable strategy for delaying bloom than increasing base temperature for GDH accumulation.

## Introduction

Peach floral buds are cold hardy during endodormancy, but lose cold hardiness during bud burst and bloom after which open flowers and developing fruit are highly sensitive to freezing temperatures (Lang, 1987). Postbloom, freezing exposures as short as a few hours can result in partial to total loss of the annual crop (Bassi and Monet, 2008). The interval between floral bud break and the last frost of the spring is, therefore, a critical period of risk exposure to producers.

Two temperature requirements act to determine floral bud break and avoid potentially lethal freezing temperatures in the spring. Peach floral buds must experience a quantitative exposure to chilling temperatures [chilling requirement (CR)] to allow bud development to proceed in response to quantitative accumulation of warm temperatures [heat requirement (HR)]. Variety CR has long been recognized as a major determinant of bloom timing, with breeding for later bloom dates resulting in selection for increased CR (Topp et al., 2008).

Producers are constrained in cultivar use by the reliable minimum chilling accumulation expected in their location (Campoy et al., 2019). Planting cultivars whose CR is not met results in disrupted development or in low or no fruit set (Li et al., 2016). Conversely, planting low CR cultivars, which are certain to have their chilling fulfilled, will guarantee bloom occurs, but this bloom is easily triggered during a short period of warm weather (a “false spring”) that greatly increases the risk of crop loss from a subsequent return to freezing conditions (Chen et al., 2016; Chamberlain et al., 2019). As the first environmental hurdle to bloom, genetic diversity in CR has received the most attention in the scientific literature, while potential genetic diversity in HR has been relatively unexplored (Topp et al., 2008; Bielenberg et al., 2015; Anzanello and Biasi, 2016).

Breeding for floral bud HR may be an unexploited mechanism for adapting peach trees to avoid exposure to freezing conditions and reduce crop losses following chilling fulfillment, but has received less attention due to the methodological difficulty of estimating variety HR from field experiments (Okie and Blackburn, 2011a,b). HR is the quantitative thermal time, which must be accumulated to reach the developmental stage of bud break. Thermal time is often described in units of degree days or degree hours (i.e., °C⋅d or°C⋅h, respectively). Interpretation of thermal time for development requires a description of the relationship between developmental rate and different temperatures. Development rate increases between a minimum threshold temperature, i.e., base temperature (Tb) and an optimum temperature (To), while development rate decreases from the optimum temperature to a critical maximum temperature (Tc) (Trudgill et al., 2005). A linear relationship between the development rate and temperature between cardinal temperatures (Tb to To and To to Tc) has been observed in many poikilothermic species and is commonly seen in seed germination studies (Alvarado and Bradford, 2002; Trudgill et al., 2005; Okie and Blackburn, 2011a,b). However, curvilinear relationships are also observed in studies on temperature-driven plant development, based upon a variety of sigmoidal-shaped functions (Yan and Hunt, 1999; Shafii and Price, 2001).

Despite recognition that cardinal temperatures vary between individuals or populations in many plant species, information on whether this is also true in peach is lacking. Efforts to quantify variety HRs among *Prunus* spp. have generally made use of a heat accumulation response curve developed in the 1980s for *Prunus cerasus* “Montmorency” with an assumed Tb of 4.4°C (Anderson et al., 1986). The model for estimating completion of rest in peach trees proposed by Richardson et al. (1974) has recently been used to map HR in segregating peach progenies revealing major quantitative trait locus (QTL) for delaying of bloom by increasing HR (Cirilli et al., 2021). These efforts to phenotype HR have assumed that the cardinal temperatures and the response curves do not vary genetically, with differences between species/cultivars deriving only from differences in required quantitative thermal time. Anzanello and Biasi (2016) demonstrated that this assumption may not hold with their observation that Tb for vegetative bud break in two selected cultivars of peach differed considerably (2.2 vs. 6.3°C). However, floral bud break was not examined in that study (Anzanello and Biasi, 2016). Similar situation where the most common models use the same parameters in CR quantifications for all the species and cultivars is recently discussed by Egea et al. (2020) and Luedeling et al. (2021). We hypothesize that the use of the same HR model considering the same Tb for all the species and cultivars may provide insufficient information, as there is evidence of genetic diversity for both traits.

Therefore, the aim of this study was to determine whether there may be unrecognized diversity in peach germplasm for two HR traits (Tb and thermal time), which could inform breeding efforts to delay floral bud break and reduce the frost exposure risk of developing flowers and fruits.

## Materials and Methods

### Plant Material

A total of 54 peach tree accessions grown at the Clemson University Musser Fruit Research Center (latitude: 34.639038, longitude: −82.935244, and elevation: 210 m.a.s.l) were selected for the experiment. In total, 48 of these accessions were siblings from a segregating F2 population resulting from selfing of an F1 seedling (01-06245) obtained from a cross between high (“Hakuho”) and low (“UFGold”) CR parents. These individuals have been extensively phenotyped for bloom characteristics and show wide variation in CR and bloom date (Bielenberg et al., 2015). Three additional commercial cultivars (Contender, Elberta, and JuneGold) were included because of previous use in phenology modeling (Schwartz et al., 1997). Trees were at least 5 years old and grown on Guardian^®^ rootstock and trained to a perpendicular V canopy structure.

### Sample Collection, Chilling, and Warm Forcing Treatment

A total of 25 50 cm long, current year stem segments were collected from each tree (approximately 1 cm in diameter) when 105 h below 7.2°C had accumulated in the field (17 November 2018) and buds were assumed to be endodormant. Most trees had fully abscised leaves at sampling, but some retained the most apical leaves, which were removed at collection. Stems from each tree were tied in a bundle and wrapped in plastic bags with moist paper towels to avoid desiccation. Plastic bags with stems were placed in a temperature-controlled chamber without light at 3°C to provide chilling accumulation.

Chilling saturated stems were removed from the 3°C treatment at approximately 1,560 h of chilling accumulation. This duration of chilling treatment was selected to ensure that all the trees had fully saturated their chilling requirement to at least 1.5× their previously determined CR (Bielenberg et al., 2015) to avoid the confounding effects of insufficient chilling accumulation on HR (Harrington et al., 2010; Okie and Blackburn, 2011a).

Stems were trimmed to approximately 40 cm in length (removing terminal segments) and buds from the lower third of the stem removed as these would potentially be submerged. Five stems from each variety were placed in each of five containers of 2 × strength Floralife^®^ Crystal Clear [Floralife, Walterboro, South Carolina (SC), United States]. One set of five stems was placed in each of five temperature-controlled chambers for warm forcing at temperature set points of 12, 14, 16, 18, or 20°C with a 12-h photoperiod provided by red light-emitting diodes (LEDs) (Okie and Blackburn, 2011b). Temperature chambers used in this experiment were described by Bielenberg and Gasic (2019). Observed average chamber temperature was measured with dataloggers as 11.8, 13.6, 15.9, 17.8, and 20.1°C and these values were used in subsequent calculations. Temperature levels were selected to be above temperatures (9.1^°^C) at which additional chilling could accumulate by the Utah model (Cesaraccio et al., 2004) and below the assumed optimal temperature of 25°C (Anderson et al., 1986).

### Bud Break Observation

Total initial floral bud numbers were counted for each temperature × accession combination, taking advantage of the “triple bud” arrangement of peach where two floral buds flank a central vegetative bud at each node. Total floral bud number per temperature × accession combination ranged between 100 and 150. Bud break progress was observed 3 times per week, starting 24 h after experiment setup, until no changes occurred for three consecutive observations. Buds were considered open when bud scales were separated and patch of coloration (sepals or petals) was visible (stage 5, inflorescence emergence, BBCH 51) (Lancashire et al., 1991). Open floral or vegetative buds were removed on each observation date to reduce water and carbohydrate demand on the stem from floral or leaf expansion and enhance stem longevity. Floral bud break fraction was calculated as the cumulative number of buds opened relative to the initial number of floral buds present on the stems.

### Calculation of Apparent Tb and Thermal Time for Bud Break

At each temperature, hours of forcing to reach 0.5 bud break fraction were calculated by linear interpolation between the two observations, which bracketed the 0.5 value. If the bud break fraction of an accession and forcing temperature combination did not reach 0.5 and the bud fractions were no longer changing, it was not included for further analysis. A linear regression was fitted to the relationship between forcing temperature and development rate (hours^–1^ to reach 0.5 bud break fraction). The *X*-axis intercept and slope of this regression were used to determine the apparent Tb and thermal time, respectively, using the equations below.


(1) $$D=\frac{\left(T-T\,b\right)}{t\,h\,e\,r\,m\,a\,l\,t\,i\,m\,e}$$

(2) apparent⁢Tb=x(y=0)=-bm=C°

(3) $$\mathrm{thermal}\,\mathrm{time}=\frac{1}{m}{=}^{{}^{\circ}}C\cdot h$$

where, *b* = the *Y*-axis intercept and m = the slope of the linear relationship between forcing temperature and development rate (hours^–1^ to reach 0.5 bud break fraction). The term apparent Tb is used here because in all the cases, the *X*-axis intercept value was outside of the range of the values used to create the regression and was not directly observed. The Equation 1 was used to relate linear development rate and temperature.

### Simulation of Bud Break Dates From Historical Weather Data

To investigate if adjusting Tb or thermal time [growing degree hour (GDH)] would provide better opportunity to manipulate heat requirement in peach in the southeast United States, we obtained complete temperature records spanning 1989 through July 2019 from the United States National Oceanic and Atmospheric Administration (NOAA) and National Centers for Environmental Information “Integrated Surface Database” using the “rnoaa” package in R (Smith et al., 2011; Chamberlain, 2019; R Core Team, 2019). Stations KABY (Southwest Georgia Regional Airport, Albany, GA, United States; 31.53556°, −84.19444°), KGSP (Greenville–Spartanburg International Airport, Greer, SC, United States; 34.8842°, −82.2209°), and KRDU (Raleigh–Durham International Airport, Morrisville, NC, United States; 35.8923°, −78.7819°) were selected for the completeness of their records and position along a rough southwest to northeast transect along which commercial peach production occurs and to represent contrasting winter climate regimes, from warmest to coldest, respectively (Supplementary Figures 1, 2 and Supplementary Table 1). Mean hourly temperatures were used to calculate chilling accumulation from 01 October, which is typically used in the eastern United States, of each winter using the simple accumulated hours <7.2°C model of Weinberger (1950). Thermal time accumulation was calculated as the running total accumulation of the hourly difference between a minimum Tb and the observed temperature with observed temperatures below the Tb resulting in zero accumulation.

The hypothetical impact of different Tb and thermal time trait combinations on simulated bud break timing was assessed by comparing five HR trait combinations (7.3°C, 3,000°C⋅h; 4.2°C, 5,000°C⋅h; 2.2°C, 7,000°C⋅h; 0.6°C, 9,000°C⋅h; and −0.6°C, 11,000°C⋅h) that fell on the line fit to the apparent Tb and thermal time data of our measured accessions (Figure 3) to the average Tb and thermal time across locations, 2.2°C, 7,000°C⋅h. Heat accumulation and date of completion of thermal time were evaluated for each location from the day on which 500, 750, or 1,000 chilling hours had accumulated in each year to assess whether the HR trait effects would be sensitive to differences in chilling requirements among accessions. Chilling accumulation reached 1,000 h in only five of the 30 years at the KABY location.

## Results

### Bud Break and Tb

**FIGURE 1:**
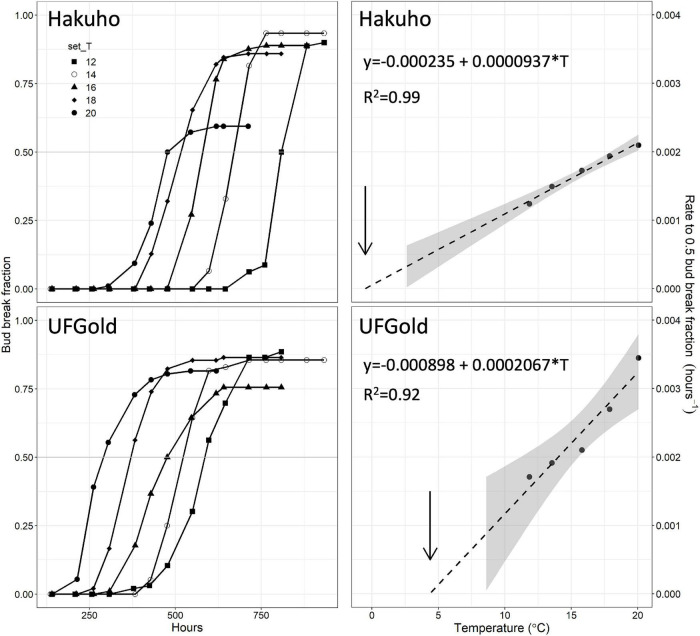
Bud break fraction with warm forcing time **(left panels)** in ‘Hakuho’ or ‘UFGold’ at five constant temperatures. Rate to 0.5 bud break fraction **(right panels)** at five temperatures calculated from data on the left in ‘Hakuho’ or ‘UFGold.’ Dashed lines are linear regressions extended to the *X*-axis intercept (*y* = 0). Arrows indicate apparent base temperature (Tb). Gray area represents 95% CI.

Bud break fraction with time followed a sigmoidal curve shape in each temperature and accession, as shown for “Hakuho” and “UFGold” in Figure 1. Bud break was delayed in each successively lower temperature (Figure 1). Maximum bud break fraction for 52 accessions ranged between 0.83 and 1.0. Two accessions, “Contender” and C021, did not reach 0.5 bud break fraction in any temperature treatment and were not included in further analysis.

**FIGURE 2:**
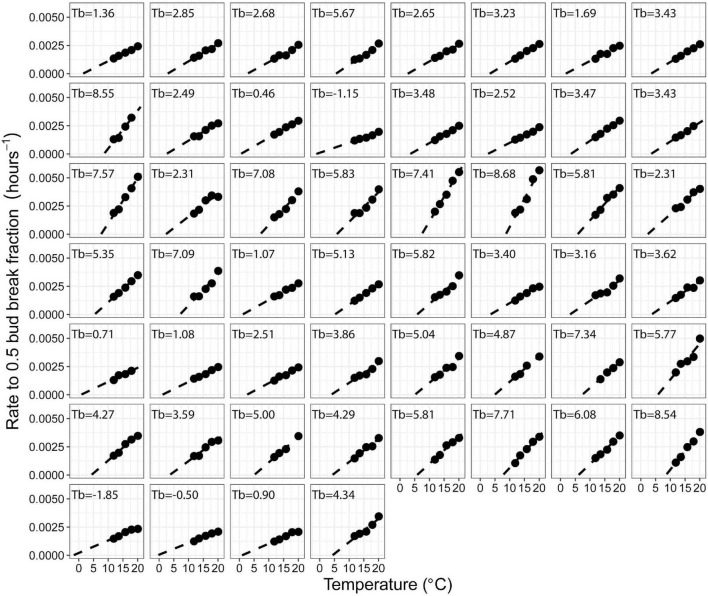
Effect of temperature on bud break rate (h^– 1^ to 0.5 bud break fraction) in warm forced cuttings of 52 peach accessions. The first 48 panels show F2 siblings from a population segregating for chilling requirement and bloom time (Bielenberg et al., 2015). The final four panels are ‘Elberta,’ ‘Hakuho,’ ‘Junegold,’ and ‘UFGold,’ respectively.

Bud break rate (hours^–1^ to 0.5 bud break fraction) linearly responded to temperature in all the accessions with a mean *R*^2^ of 0.96 (Figures 1, 2 and Supplementary Table 2).

**FIGURE 3:**
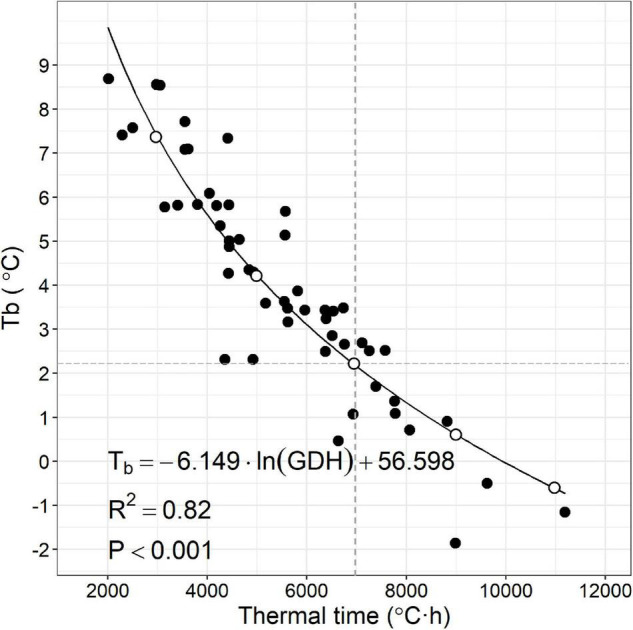
Scatterplot of apparent base temperature (Tb) and estimated thermal time (°C⋅h) of 52 peach accessions and logarithmic relationship. Gray dashed lines represent Tb (2.2) and thermal time (7,000) average observed in the data and used as a reference. White circles designate heat requirement trait combinations (Tb, thermal time) from **(left)** to **(right)**: 7.3, 3,000; 4.2, 5,000; 2.2, 7,000; 0.6, 9,000; and –0.6, 11,000.

Extrapolation of the linear relationship between bud break rate and temperature to the temperature at which no bud break would occur [x_(y = 0)_] was used to estimate the apparent Tb (Figures 1, 2).

Apparent Tb ranged from −1.85 to 8.69°C (Figure 3). Thermal time to 0.5 bud break was calculated for each accession and ranged from 2,015 to 11,191°C⋅h (Figure 3). Apparent Tb appeared to be inversely correlated to thermal time across the accessions evaluated in a logarithmic fashion (Figure 3). Low correlation was found between accession CR and accession apparent Tb or thermal time (Supplementary Figure 3).

### Impact of Different Tb and Thermal Time Trait Combinations on Simulated Bud Break

**FIGURE 4:**
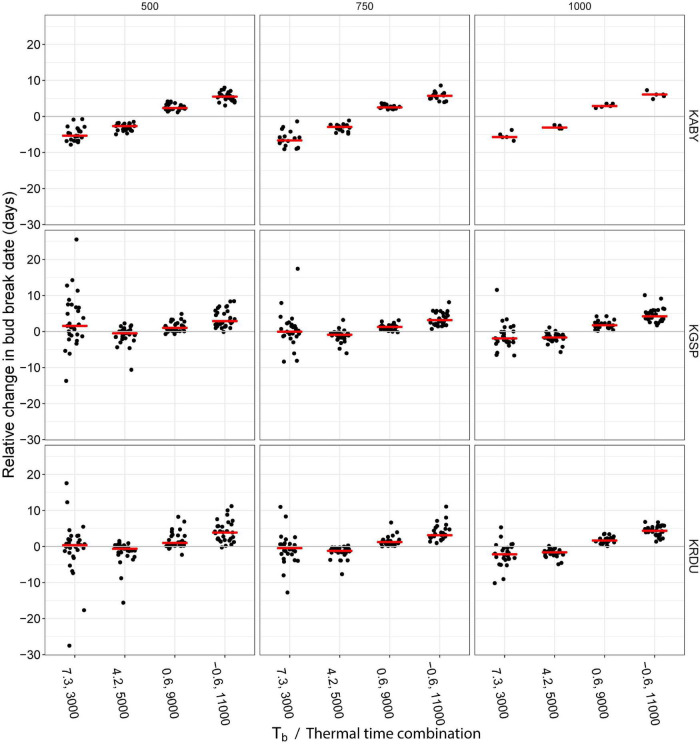
Simulation of 0.5 floral bud break fraction advance or delay following different chilling accumulation at three locations in the southeastern United States. Heat accumulation was calculated following 500 **(left panels)**, 750 **(center panels)**, or 1,000 **(right panels)** chill hour accumulation (hours < 7.2°C) at three NOAA weather stations [KABY, Georgia (GA), United States; KGSP, South Carolina (SC), United States; KRDU, North Carolina (NC), United States]. Data were shown only for years in which location reached the chilling requirements shown in figure (e.g., 500, 750, and 1,000). Red horizontal lines indicate the median days of change in bud break.

The assessment of hypothetical impact of different Tb and thermal time trait combinations on simulated bud break timing revealed shifting of HR traits to a lower Tb and higher thermal time than the reference 2.2°C, 7,000°C⋅h combination and a delay of simulated bud break date in all the locations and for each chilling accumulation (Figure 4). Significant differences between evaluated Tb thermal time combinations and reference were observed for all the combinations, except 7.3^°^C, 3,000°C h combination at 1,000 chill hours (CH) at KGSP location, at 750 CH at KGSP and KRDU locations, and at 500 CH at KRDU location (Supplementary Table 3). While this delay in simulated bud break was as much as 11 days in one location and year combination (KRDU/1999), the median delay in the −0.6°C, 11,000°C⋅h combination was between 3 and 6 days by location and chilling hour accumulation. Shifting HR traits to a higher Tb and lower thermal time resulted in a nearly symmetrical advance of simulated bud break dates at the KABY location relative to the lower Tb and higher thermal time. When HR traits were shifted to higher Tb and lower thermal time at the KGSP and KRDU locations, simulated relative bud break dates became highly variable among years with as much as a 27.5-days advance (KRDU, 500 chill hours) or a 25.5-days delay (KGSP, 500 chilling hours) in bud break (Figure 4). Despite the larger variation in simulated bud break dates, the simulated median bud break for the 7.3°C, 3,000°C⋅h combination among years only differed from the 2.2°C, 7,000°C⋅h combination by 1 day either advanced or delayed (Figure 4).

**FIGURE 5:**
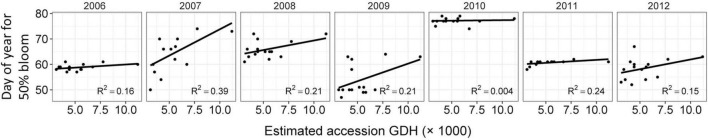
Effect of °C⋅h to bud break compared to the day of 50% bloom in the field across 7 years (2006–2012). Data represent field bloom day of fourteen accessions with estimated 650–750 chilling hour requirements, but differing thermal times for 0.5 bud break fraction. Slopes of linear regression (*y = mx + b*) analyses were not significantly different (*P* < 0.001) from zero except for 2007.

A total of 50 accessions in this study have 7 years of common garden bloom data (2006–2012) available from a previous study where their chilling requirement was also determined (Bielenberg et al., 2015). In total, 14 of these 50 accessions have a chilling requirement estimated to be between 650 and 750 chilling hours (Bielenberg et al., 2015). Apparent Tb and thermal times of these fourteen accessions ranged from 1.3 to 7.1°C and 3,405 to 7,766°C⋅h, respectively, potentially allowing observation of effects of HR trait variation on bloom date, while minimizing the effect of variety differences in CR. Effect of the estimated thermal time (°C⋅h) to bud break determined in this study was compared to the day of 50% bloom in the field across 7 years (Figure 5) with the assumption that variety differences in GDH to reach 0.5 bud break would still be present at 50% of full bloom. Spread in bloom dates differed by year with < 10 day spread in 2006, 2010, and 2011 and 15–25 day spreads in the other years (Figure 5). There was a trend of delayed 50% bloom with increasing thermal time (°C⋅h) of accession. However, slopes of the linear regressions between thermal time and day of 50% bloom were not significantly different from zero except for 2007.

## Discussion

Diversity in HR for floral bud break among peach cultivars (and other *Prunus* spp.) has been assumed to primarily be based on different thermal time requirements calculated from a common base temperature (Okie and Blackburn, 2011a; Maulión et al., 2014). Our results demonstrate that this assumption is not justified (Figure 3). Cultivar differences in apparent Tb and thermal time requirement could provide additional sophistication to improve models of bloom timing in the spring for grower decision-making.

While we have highlighted an important area of unrecognized diversity in heat requirement behavior in peach, we should note that the methods used in this study have two important caveats. First, we have termed our Tb values as “apparent Tb” because they are extrapolations below our lowest observed forcing temperature of 12°C. It is possible that the linearity of our forcing data does not hold to the extrapolated Tb values. Our selection of temperatures was driven by following concerns: (A) even though we provided excess chilling prior to the experiment, we wished to avoid temperatures where both the chilling and heat accumulation can occur simultaneously; (B) our relatively “warm” temperatures caused faster development and lowered the risk of losing stem cuttings to blockages of water transport and desiccation during prolonged forcing; and (C) at the lower temperatures (<10^°^C), stem cuttings would have to be kept longer, which would reduce our ability to keep them alive and prevent flower opening due to stem death and not to the experimental treatment. Even at 12°C, some accessions in this study required more than 50 days to complete floral bud break and some never reached 0.5 bud break fraction in any temperature treatment. The reason for some accessions not being able to reach 0.5 bud break fraction is unclear and needs to be further researched. We suspect that it is biological and associated with inability to tolerate extended period of maintenance as cuttings in water. Working with leaf buds (which are more resilient to forcing duration survival and can be maintained for >100 days), Anzanello and Biasi (2016) showed a positive linear relationship between temperature and bud break from 0 to 10°C across several species, which suggests our linear extrapolation may not be unfounded. Assuming linear positive relationship between temperature and bud break, we clearly show that there were different response curves for the evaluated cultivars, which supported our hypothesis of existing genetic diversity that could be exploited in breeding. Despite uncertainty in determining Tb under temperature regimes used in this study and discussed above, the observed differences in apparent Tb in peach germplasm included in this study clearly support our hypothesis. In addition to the study by Anzanello and Biasi (2016) on Tb of peach, plum, grape, pear, and kiwi, similar evidence of differences in Tb among cultivars within a single fruit species, apple, was also reported (Putti et al., 2000; Anazanello, 2012).

Second, we have likely underestimated the thermal time requirements of the eighteen accessions whose apparent Tb falls below 3°C. We designed our experiment to avoid an overestimation of thermal time that can result when buds have only received the minimum chilling to be competent to break (Harrington et al., 2010; Okie and Blackburn, 2011a). Stem cuttings of all the accessions were chilled at 3°C for 65 days (approximately 1,560 h). CR of accessions with apparent Tb below 3°C ranged from 400 to 1,000 chill hours, a duration which would be between 1.5× and 4.0× the estimated minimal chilling requirement of these accessions. The further below 3°C the Tb of the accession, the greater degree of underestimation of the thermal time should be expected. Accessions with lower CR will also have a greater degree of underestimation; due to the increased length of time; they would have been accumulating heat following chilling satisfaction. Our thermal time values for these accessions should; therefore; be considered as minimums. The curvilinear relationship between thermal time and apparent Tb below 3°C (Figure 3) should, therefore, be used with caution.

We observed an unexpected degree of variation in both the apparent Tb and thermal time. It should be noted that 48 of the accessions we observed are F2 siblings derived from a self-pollinated F1 individual resulting from a cross between high CR and low CR parents (Bielenberg et al., 2015). Therefore, much of the observed variation arose from the diversity of loci/alleles present in that single F1 individual. Even though plant material evaluated in this study included accessions known to differ in CR and bloom time, we had no *a priori* reason to suspect significant variation in HR traits. The full extent of the phenotypic diversity in the species (Monet and Bassi, 2008) has not been assayed here, as thousands of peach cultivars exist from breeding programs in many countries and climates. The range of Tb and thermal time found in these few accessions suggests that there may be enough diversity in HR traits to potentially include HR traits as a selection trait in breeding programs. A critical first step in this process will be screening material in germplasm repositories to assess the full extent of diversity available.

The negative relationship between apparent Tb and thermal time (Figure 3) poses a conundrum about which trait would be the most profitable to manipulate for the goal of delayed bloom time. Increasing either Tb or thermal time requirement would result in delayed bud break. However, the negative correlation between the two traits suggests increasing one cannot be achieved without decreasing the other. Assuming the negative relationship between apparent Tb and thermal time is the result of an underlying linkage (genetic or mechanistic) in the traits, the choice of a desirable phenotype for new cultivars is not obvious. We, therefore, investigated the bud break behavior of trees with five different hypothetical combinations of HR traits representing different positions along the line fitted to the 52 combinations of thermal time and apparent Tb observed in this study using historical weather data (Figure 4).

While increasing apparent Tb alone was expected to delay bloom, the correlated decrease in thermal time actually resulted in accelerated bloom dates, resulting in advanced bloom at the warmer GA location and highly variable but, on average, unchanged bloom timing in the SC and NC locations (Figure 4). The significant advances in simulated bud break date observed at the warmest location, KABY, could be due to warm temperatures before complete endodormancy release boosting the dormancy-breaking process, as suggested by Harrington et al. (2010). The advance of bloom in approximately half of the years (in SC or NC) when thermal time requirement is reduced would increase bloom/fruit exposure to potential frost exposure, dramatically increasing risk of crop loss. Increasing thermal time requirement appeared to consistently delay bloom time in all the three locations, despite the expected increase in daily thermal time accumulation resulting from a decreased Tb (Figure 4). The non-linear shape of the correlation between apparent Tb and thermal time likely explains this asymmetry of response, as apparent Tb is not decreasing as quickly as thermal time requirement is increasing at the high thermal time portion of the relationship. Our underestimation of thermal time requirement in accessions with apparent Tb values below 3°C noted above means that we have underestimated the delaying effect shown here.

Acknowledging the fact that our conclusion might be heavily biased due to the small number of peach varieties and type of the material used in this study (individuals from a single cross), we propose that focusing on increased thermal time for bud break of peach would be a more reliable strategy to delay bloom than increasing the Tb for thermal time accumulation in the southeastern United States. In addition, chill hours model used in this study, due to its simplicity, is not the most suitable for warm areas such as KABY location, as negation effect produced by warm temperatures is not captured. Other models such as Dynamic and or Utah models would be more appropriate to capture them and provide more refined results (Benmoussa et al., 2017). However, this study has focused on diversity of heat requirement traits in peach germplasm as less researched traits and deliberately did not expand on chilling accumulation. Even with all the flaws of chill hour model, °C⋅h shows diversity in the peach germplasm that is available for breeding. As chilling requirement of fruit cultivars, including peach, is provided in chill hours (Gasic et al., 2020), there is a need to also provide a chill portions to allow researchers and growers to more accurately predict climate effect on fruit tree production.

It remains to be determined if the correlation between apparent Tb and thermal time requirement can be broken. If apparent Tb and thermal time requirement are not closely genetically linked, it could be possible to independently manipulate them. Our observations suggest that there is some latitude to alter one without affecting the other (Figure 3). It is also possible that there is a yet unknown physiological mechanism linking these two characters, which would not allow them to be independently selected. Negative correlations between thermal time and base temperature for developmental milestones in plants and other poikilothermic species have been observed (Trudgill et al., 2005), raising the possibility that there may be a fundamental mechanistic linkage between these two traits.

Since CR has a profound effect on bloom date before HR traits can be expressed, it is difficult to observe the effects of variation in HR traits in the field. In an attempt to connect estimated GDH to field behavior and assuming that variety differences in GDH to reach 0.5 bud break would still be present at 50% full bloom, we have observed effects of HR trait variation on bloom date in 14 siblings with similar CR. Even though we have no information on whether GDH from bud break to bloom radically changes in a variety-dependent fashion in a different pattern than that from dormant to bud break, we found some evidence that increased thermal time requirement results in a greater likelihood of delayed bloom across multiple years (Figure 5). The delayed bloom behavior was evident despite the variation in CR (between 650 and 750 h < 7°C) and apparent Tb (−1.1 to 6°C) of these accessions, which could mask the effect of variation in thermal time requirement. Despite issues in estimating Tb discussed above, the observed large differences in thermal time requirement between individuals from a single cross show that there is a possibility to distinguish the responsiveness of an individual to temperature/forcing. These observations support our hypothesis that breeding for increased thermal time requirement could be a viable strategy to delay bloom and avoid exposure to potential crop destroying frosts. In a future of decreasing winter chilling, but continued possibilities of short duration frost events, delaying bloom through altered HR traits could be a valuable means of increasing resilience in peach or other spring blooming perennial crops. The information on genetic diversity of HR traits in peach germplasm could potentially also be used to advance bloom time in environments where late spring frosts are not an issue for production and in combination with reduced CR, to target certain ripening windows and position the product in the market.

Overall, this study can be considered as a first attempt to find out two important aspects of temperate fruit tree phenology: genetic variation of HR traits and their possible relationship with CR, if there is any. There are still many uncertainties and more experimentation and data analyses must be carried out. As discussed earlier, the methodology for estimating Tb and GDH from experiments should be improved. The results of these experiments could help in driving breeding programs toward development of new cultivars that adapt to novel scenarios of climate change by combining low CR and high HR to minimize frost risk.

## Data Availability Statement

The raw data supporting the conclusions of this article will be made available by the authors, without undue reservation.

## Author Contributions

DB and KG contributed to the conceptualization, funding acquisition, resources, supervision, and writing—review and editing. Both authors have read and approved the final manuscript.

## Conflict of Interest

The authors declare that the research was conducted in the absence of any commercial or financial relationships that could be construed as a potential conflict of interest.

## Publisher’s Note

All claims expressed in this article are solely those of the authors and do not necessarily represent those of their affiliated organizations, or those of the publisher, the editors and the reviewers. Any product that may be evaluated in this article, or claim that may be made by its manufacturer, is not guaranteed or endorsed by the publisher.

## Supplementary Material

Supplementary Figure 1 Mean daily **(A)** and monthly **(B)** temperatures observed at the three stations KABY [Albany, GA, United States; 31.53556^°^, −84.19444^°^], KGSP [Greer, SC, United States; 34.8842^°^, −82.2209^°^], and KRDU (Morrisville, NC, United States; 35.8923^°^, −78.7819^°^] during 1989–2019 within the chill accumulation period of 01 October to 28 February. Data represent averages calculated from the hourly temperatures obtained for each day in each month. Only years for which data for all the three stations were available were shown. Mon.Day—month and day.

Supplementary Figure 2 Chill accumulation at the three stations KABY (Albany, GA, United States; 31.53556^°^, −84.19444^°^), KGSP (Greer, SC, United States; 34.8842^°^, −82.2209^°^), and KRDU (Morrisville, NC, United States; 35.8923^°^, −78.7819^°^) during 1989–2019. Data represent total chill hours **(A)** and chill portions **(B)** accumulated within the chill accumulation period of 01 October to 28 February. Only years for which data for all the three stations were available were shown.

Supplementary Figure 3 Correlation between chilling requirement (CH), base temperature (Tb), and thermal time [growing degree hour (GDH)] in F2 siblings from a population segregating for chilling requirement and bloom time (Bielenberg et al., 2015) and four cultivars such as Elberta, Hakuho, Junegold, and UFGold, respectively.
