# Supplementary figures and images for: Peach [Prunus persica (L.) Batsch] Cultivars Differ in Apparent Base Temperature and Growing Degree Hour Requirement for Floral Bud Break
Source: Front Plant Sci. 2022 Feb 11;13:801606. doi: 10.3389/fpls.2022.801606 (PMC8874129; doi:10.3389/fpls.2022.801606)

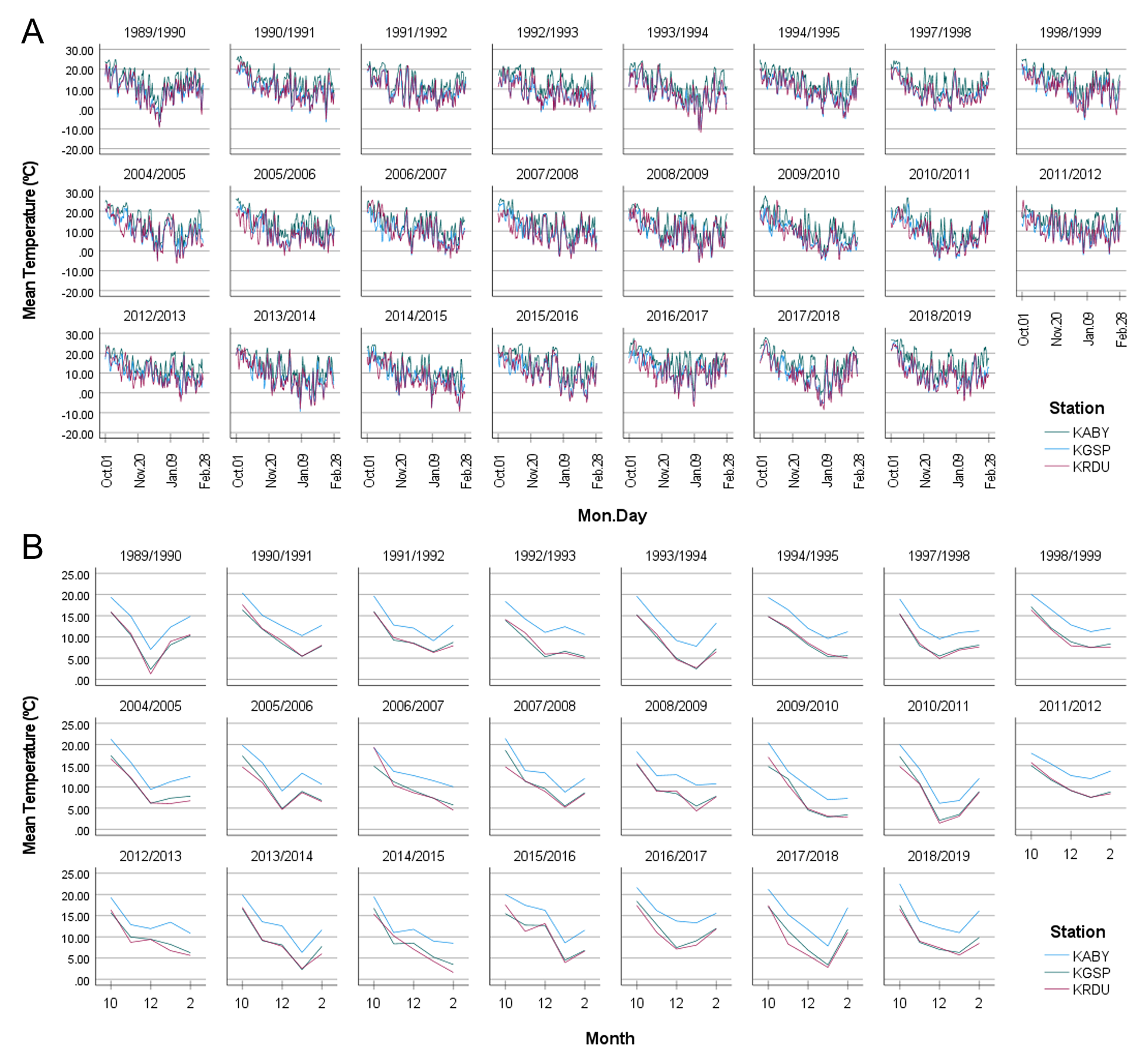

Supplement: Supplementary Figure 1 — Mean daily (A) and monthly (B) temperatures observed at the three stations KABY [Albany, GA, United States; 31.53556°, −84.19444°], KGSP [Greer, SC, United States; 34.8842°, −82.2209°], and KRDU (Morrisville, NC, United States; 35.8923°, −78.7819°] during 1989–2019 within the chill accumulation period of 01 October to 28 February. Data represent averages calculated from the hourly temperatures obtained for each day in each month. Only years for which data for all the three stations were available were shown. Mon.Day—month and day. [file Image_1.TIF]

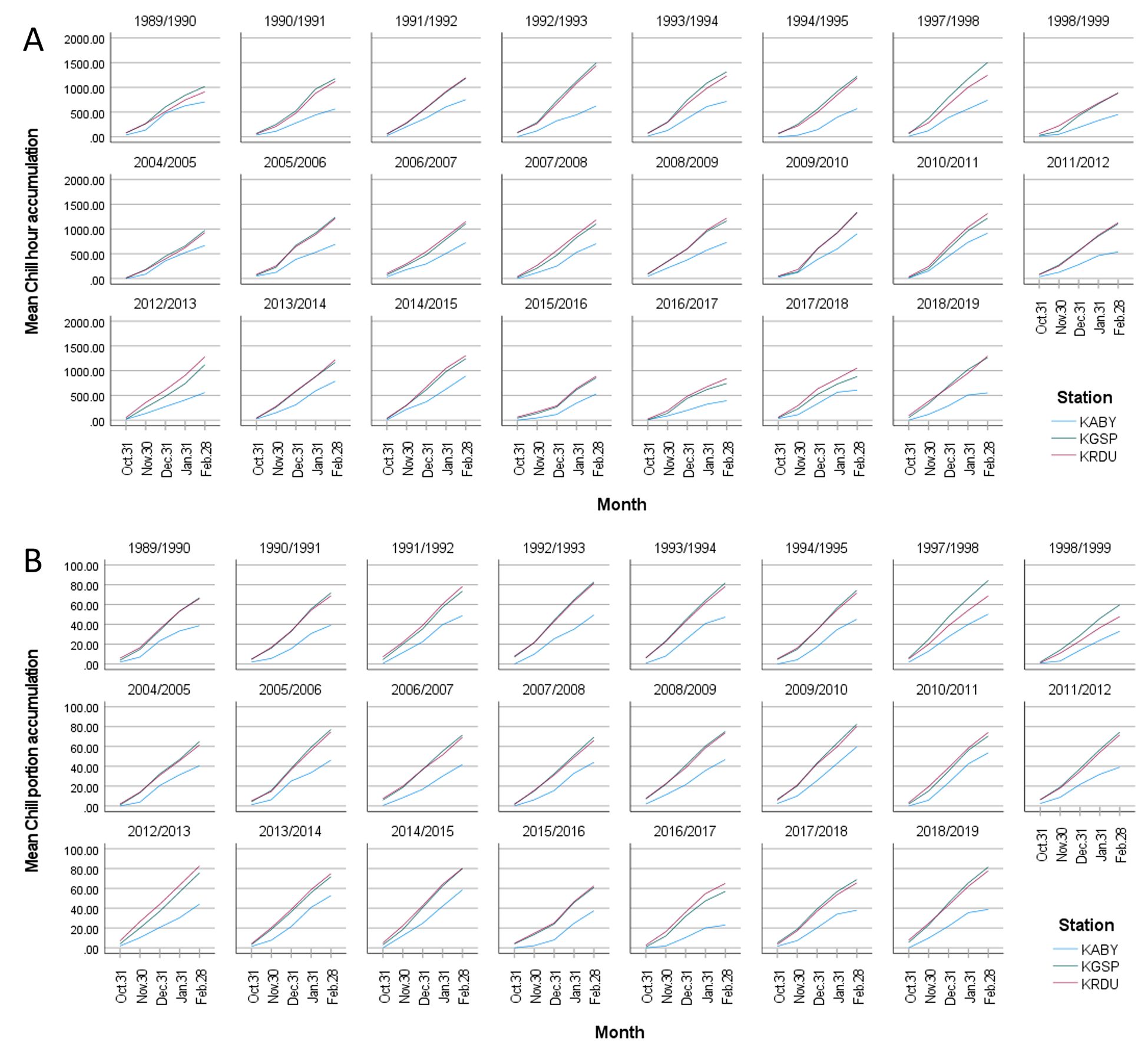

Supplement: Supplementary Figure 2 — Chill accumulation at the three stations KABY (Albany, GA, United States; 31.53556°, −84.19444°), KGSP (Greer, SC, United States; 34.8842°, −82.2209°), and KRDU (Morrisville, NC, United States; 35.8923°, −78.7819°) during 1989–2019. Data represent total chill hours (A) and chill portions (B) accumulated within the chill accumulation period of 01 October to 28 February. Only years for which data for all the three stations were available were shown. [file Image_2.TIF]

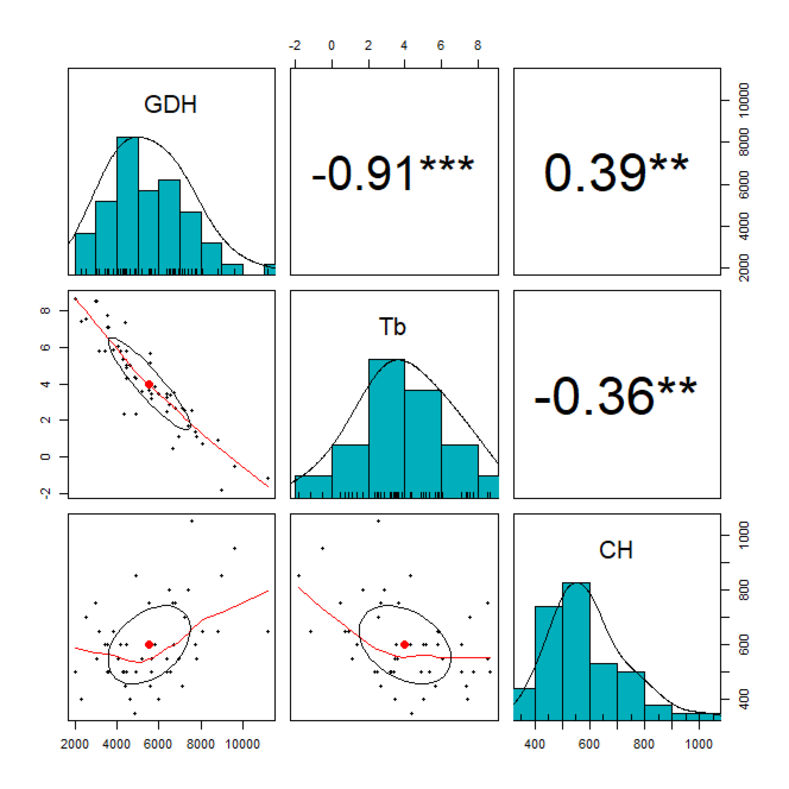

Supplement: Supplementary Figure 3 — Correlation between chilling requirement (CH), base temperature (Tb), and thermal time [growing degree hour (GDH)] in F2 siblings from a population segregating for chilling requirement and bloom time (Bielenberg et al., 2015) and four cultivars such as Elberta, Hakuho, Junegold, and UFGold, respectively. [file Image_3.TIF]
