# Supplementary material for: Peach [Prunus persica (L.) Batsch] Cultivars Differ in Apparent Base Temperature and Growing Degree Hour Requirement for Floral Bud Break
Source: Front Plant Sci. 2022 Feb 11;13:801606. doi: 10.3389/fpls.2022.801606 (PMC8874129; doi:10.3389/fpls.2022.801606)
Supplement: Supplementary file 4 [file Table_1.docx]

Supplementary Table 1. Chilling requirement, base temperature (Tb), thermal time (GHD) and coefficient of determination (R2) between base temperature and thermal time for 48 F2 siblings from population segregating for chilling requirement and bloom time (Bielenberg et al., 2015) and four cultivars.

| Accession | CH | Tb | GDH | R2 |
| --- | --- | --- | --- | --- |
| c01 | 600 | 1.36203832 | 7766.5606 | 1 |
| c02 | 800 | 2.85288019 | 6514.22441 | 0.97 |
| c04 | 450 | 2.6892569 | 7113.68423 | 0.92 |
| c05 | 450 | 5.67336822 | 5566.42511 | 0.95 |
| c07 | 750 | 2.65797307 | 6759.88188 | 0.97 |
| c08 | 500 | 3.23644983 | 6385.49716 | 1 |
| c09 | 800 | 1.69921766 | 7389.00208 | 0.93 |
| c11 | 550 | 3.43212056 | 6368.44467 | 1 |
| c12 | 750 | 8.55372338 | 2977.42889 | 0.96 |
| c13 | 500 | 2.49274149 | 6370.63428 | 0.96 |
| c14 | 750 | 0.4657037 | 6635.84049 | 1 |
| c15 | 650 | -1.1533561 | 11191.2398 | 0.96 |
| c17 | 600 | 3.48763379 | 6736.84715 | 0.99 |
| c18 | 1050 | 2.5205247 | 7577.3487 | 0.99 |
| c19 | 550 | 3.47848807 | 5614.47278 | 1 |
| c20 | 400 | 3.43418986 | 5961.91639 | 0.99 |
| c22 | 700 | 7.57186276 | 2498.10633 | 0.99 |
| c23 | 500 | 2.31111838 | 4920.84554 | 0.88 |
| c24 | 600 | 7.08790301 | 3545.04463 | 0.97 |
| c25 | 650 | 5.83437018 | 3804.78089 | 0.92 |
| c26 | 400 | 7.41091558 | 2286.17093 | 0.99 |
| c27 | 500 | 8.68772715 | 2014.8324 | 0.96 |
| c28 | 600 | 5.81706605 | 3405.87644 | 0.97 |
| c29 | 500 | 2.31522825 | 4355.63013 | 0.97 |
| c30 | 500 | 5.35280911 | 4260.8125 | 1 |
| c31 | 500 | 7.09052514 | 3613.31123 | 0.93 |
| c34 | 550 | 1.07332959 | 6934.25957 | 0.98 |
| c35 | 600 | 5.13673777 | 5562.61117 | 1 |
| c36 | 600 | 5.82193941 | 4433.24111 | 0.92 |
| c38 | 500 | 3.40679455 | 6533.54791 | 0.98 |
| c39 | 500 | 3.16151112 | 5620.56799 | 0.9 |
| c40 | 750 | 3.6295334 | 5549.83711 | 0.94 |
| c41 | 650 | 0.71070577 | 8068.30433 | 0.92 |
| c42 | 550 | 1.08958544 | 7775.23562 | 0.99 |
| c43 | 700 | 2.51139206 | 7254.74797 | 0.98 |
| c44 | 600 | 3.86682553 | 5814.51331 | 0.91 |
| c45 | 400 | 5.04230142 | 4643.55564 | 0.93 |
| c46 | 500 | 4.87410185 | 4436.26442 | 0.98 |
| c47 | 450 | 7.34088482 | 4407.57368 | 1 |
| c48 | 650 | 5.77515496 | 3139.21046 | 0.89 |
| c49 | 600 | 4.27244885 | 4426.10267 | 0.98 |
| c50 | 550 | 3.59200923 | 5171.5354 | 0.94 |
| c51 | 600 | 5.0097706 | 4439.61769 | 0.99 |
| c52 | 850 | 4.29694742 | 4929.70214 | 0.96 |
| c53 | 600 | 5.81028737 | 4187.81314 | 0.97 |
| c54 | 500 | 7.7164394 | 3548.50937 | 0.99 |
| c55 | 550 | 6.0826961 | 4044.26439 | 0.99 |
| c57 | 550 | 8.54079425 | 3046.61466 | 1 |
| Elberta | 850 | -1.8549832 | 8985.87826 | 0.95 |
| Hakuho | 950 | -0.5039055 | 9624.61803 | 0.95 |
| Junegold | 650 | 0.90550839 | 8824.34665 | 0.96 |
| UFGold | 350 | 4.34778213 | 4838.97116 | 0.93 |
