# Supplementary material for: Peach [Prunus persica (L.) Batsch] Cultivars Differ in Apparent Base Temperature and Growing Degree Hour Requirement for Floral Bud Break
Source: Front Plant Sci. 2022 Feb 11;13:801606. doi: 10.3389/fpls.2022.801606 (PMC8874129; doi:10.3389/fpls.2022.801606)
Supplement: Supplementary file 5 [file Table_2.docx]

Supplementary Table 2. Comparison between the mean of each base temperature (Tb) thermal time (GHD) combination and overall mean (2.2ºC, 7000 ºC h) at three locations (KABY, Albany GA; KGSP, Greer SC; KRDU, Morrisville NC) in the southeastern U.S.A. Significant differences at P <0.001 (***) and 0.05 (*) determined with simple t-test.

| Location | CH | Tb | GDH | P value | Significance level |
| --- | --- | --- | --- | --- | --- |
| KABY | 500 | -0.6 | 11000 | 0.000 | *** |
| KABY | 500 | 0.6 | 9000 | 0.000 | *** |
| KABY | 500 | 4.2 | 5000 | 0.000 | *** |
| KABY | 500 | 7.3 | 3000 | 0.000 | *** |
| KABY | 750 | 7.3 | 3000 | 0.000 | *** |
| KABY | 750 | 4.2 | 5000 | 0.000 | *** |
| KABY | 750 | 0.6 | 9000 | 0.000 | *** |
| KABY | 750 | -0.6 | 11000 | 0.000 | *** |
| KABY | 1000 | 7.3 | 3000 | 0.000 | *** |
| KABY | 1000 | 4.2 | 5000 | 0.000 | *** |
| KABY | 1000 | 0.6 | 9000 | 0.000 | *** |
| KABY | 1000 | -0.6 | 11000 | 0.000 | *** |
| KGSP | 500 | 7.3 | 3000 | 0.040 | * |
| KGSP | 500 | 4.2 | 5000 | 0.039 | * |
| KGSP | 500 | 0.6 | 9000 | 0.000 | *** |
| KGSP | 500 | -0.6 | 11000 | 0.000 | *** |
| KGSP | 750 | 7.3 | 3000 | 0.632 | NS |
| KGSP | 750 | 4.2 | 5000 | 0.001 | *** |
| KGSP | 750 | 0.6 | 9000 | 0.000 | *** |
| KGSP | 750 | -0.6 | 11000 | 0.000 | *** |
| KGSP | 1000 | 7.3 | 3000 | 0.070 | NS |
| KGSP | 1000 | 4.2 | 5000 | 0.000 | *** |
| KGSP | 1000 | 0.6 | 9000 | 0.000 | *** |
| KGSP | 1000 | -0.6 | 11000 | 0.000 | *** |
| KRDU | 500 | 7.3 | 3000 | 0.632 | NS |
| KRDU | 500 | 4.2 | 5000 | 0.017 | * |
| KRDU | 500 | 0.6 | 9000 | 0.000 | *** |
| KRDU | 500 | -0.6 | 11000 | 0.000 | *** |
| KRDU | 750 | 7.3 | 3000 | 0.418 | NS |
| KRDU | 750 | 4.2 | 5000 | 0.000 | *** |
| KRDU | 750 | 0.6 | 9000 | 0.000 | *** |
| KRDU | 750 | -0.6 | 11000 | 0.000 | *** |
| KRDU | 1000 | 7.3 | 3000 | 0.001 | *** |
| KRDU | 1000 | 4.2 | 5000 | 0.000 | *** |
| KRDU | 1000 | 0.6 | 9000 | 0.000 | * |
| KRDU | 1000 | -0.6 | 11000 | 0.000 | *** |
