# Supplementary material for: Peach [Prunus persica (L.) Batsch] Cultivars Differ in Apparent Base Temperature and Growing Degree Hour Requirement for Floral Bud Break
Source: Front Plant Sci. 2022 Feb 11;13:801606. doi: 10.3389/fpls.2022.801606 (PMC8874129; doi:10.3389/fpls.2022.801606)
Supplement: Supplementary file 6 [file Table_3.docx]

Supplementary Table 3. Chill accumulation at the three stations KABY (Albany, GA, U.S.A; 31.53556°, -84.19444°), KGSP (Greer, SC, U.S.A.; 34.8842°, -82.2209°), and KRDU (Morrisville, NC, U.S.A.; 35.8923°, -78.7819°) during 1989 – 2019. Data represent total chill hours and chill portions accumulated within the chill accumulation period of October 1^st^ to February 28^th^. Only years for which data for all three stations were available were shown.

| Station | Season | Month | Chill Portions | Chill Hours |
| --- | --- | --- | --- | --- |
| KABY | 1989/1990 | Feb.28 | 39 | 706 |
| KGSP | 1989/1990 | Feb.28 | 67 | 1018 |
| KRDU | 1989/1990 | Feb.28 | 66 | 911 |
| KABY | 1990/1991 | Feb.28 | 39 | 563 |
| KGSP | 1990/1991 | Feb.28 | 72 | 1176 |
| KRDU | 1990/1991 | Feb.28 | 69 | 1122 |
| KABY | 1991/1992 | Feb.28 | 49 | 752 |
| KGSP | 1991/1992 | Feb.28 | 74 | 1183 |
| KRDU | 1991/1992 | Feb.28 | 78 | 1196 |
| KABY | 1992/1993 | Feb.28 | 50 | 624 |
| KGSP | 1992/1993 | Feb.28 | 83 | 1494 |
| KRDU | 1992/1993 | Feb.28 | 81 | 1437 |
| KABY | 1993/1994 | Feb.28 | 47 | 718 |
| KGSP | 1993/1994 | Feb.28 | 82 | 1315 |
| KRDU | 1993/1994 | Feb.28 | 78 | 1232 |
| KABY | 1994/1995 | Feb.28 | 45 | 569 |
| KGSP | 1994/1995 | Feb.28 | 74 | 1224 |
| KRDU | 1994/1995 | Feb.28 | 72 | 1188 |
| KABY | 1997/1998 | Feb.28 | 50 | 741 |
| KGSP | 1997/1998 | Feb.28 | 84 | 1504 |
| KRDU | 1997/1998 | Feb.28 | 69 | 1246 |
| KABY | 1998/1999 | Feb.28 | 33 | 452 |
| KGSP | 1998/1999 | Feb.28 | 60 | 888 |
| KRDU | 1998/1999 | Feb.28 | 48 | 884 |
| KABY | 2004/2005 | Feb.28 | 41 | 667 |
| KGSP | 2004/2005 | Feb.28 | 65 | 973 |
| KRDU | 2004/2005 | Feb.28 | 62 | 929 |
| KABY | 2005/2006 | Feb.28 | 46 | 691 |
| KGSP | 2005/2006 | Feb.28 | 77 | 1240 |
| KRDU | 2005/2006 | Feb.28 | 75 | 1214 |
| KABY | 2006/2007 | Feb.28 | 42 | 725 |
| KGSP | 2006/2007 | Feb.28 | 72 | 1109 |
| KRDU | 2006/2007 | Feb.28 | 69 | 1150 |
| KABY | 2007/2008 | Feb.28 | 44 | 702 |
| KGSP | 2007/2008 | Feb.28 | 69 | 1104 |
| KRDU | 2007/2008 | Feb.28 | 66 | 1185 |
| KABY | 2008/2009 | Feb.28 | 47 | 730 |
| KGSP | 2008/2009 | Feb.28 | 75 | 1164 |
| KRDU | 2008/2009 | Feb.28 | 74 | 1219 |
| KABY | 2009/2010 | Feb.28 | 60 | 906 |
| KGSP | 2009/2010 | Feb.28 | 83 | 1339 |
| KRDU | 2009/2010 | Feb.28 | 80 | 1335 |
| KABY | 2010/2011 | Feb.28 | 54 | 917 |
| KGSP | 2010/2011 | Feb.28 | 71 | 1219 |
| KRDU | 2010/2011 | Feb.28 | 74 | 1314 |
| KABY | 2011/2012 | Feb.28 | 39 | 538 |
| KGSP | 2011/2012 | Feb.28 | 74 | 1104 |
| KRDU | 2011/2012 | Feb.28 | 72 | 1128 |
| KABY | 2012/2013 | Feb.28 | 44 | 558 |
| KGSP | 2012/2013 | Feb.28 | 76 | 1121 |
| KRDU | 2012/2013 | Feb.28 | 83 | 1280 |
| KABY | 2013/2014 | Feb.28 | 53 | 788 |
| KGSP | 2013/2014 | Feb.28 | 72 | 1170 |
| KRDU | 2013/2014 | Feb.28 | 75 | 1223 |
| KABY | 2014/2015 | Feb.28 | 59 | 892 |
| KGSP | 2014/2015 | Feb.28 | 80 | 1243 |
| KRDU | 2014/2015 | Feb.28 | 80 | 1304 |
| KABY | 2015/2016 | Feb.28 | 37 | 529 |
| KGSP | 2015/2016 | Feb.28 | 61 | 856 |
| KRDU | 2015/2016 | Feb.28 | 63 | 889 |
| KABY | 2016/2017 | Feb.28 | 23 | 393 |
| KGSP | 2016/2017 | Feb.28 | 57 | 742 |
| KRDU | 2016/2017 | Feb.28 | 65 | 842 |
| KABY | 2017/2018 | Feb.28 | 38 | 608 |
| KGSP | 2017/2018 | Feb.28 | 69 | 881 |
| KRDU | 2017/2018 | Feb.28 | 66 | 1054 |
| KABY | 2018/2019 | Feb.28 | 39 | 552 |
| KGSP | 2018/2019 | Feb.28 | 82 | 1265 |
| KRDU | 2018/2019 | Feb.28 | 78 | 1297 |
